# FCRL5 Delineates Functionally Impaired Memory B Cells Associated with *Plasmodium falciparum* Exposure

**DOI:** 10.1371/journal.ppat.1004894

**Published:** 2015-05-19

**Authors:** Richard T. Sullivan, Charles C. Kim, Mary F. Fontana, Margaret E. Feeney, Prasanna Jagannathan, Michelle J. Boyle, Chris J. Drakeley, Isaac Ssewanyana, Felistas Nankya, Harriet Mayanja-Kizza, Grant Dorsey, Bryan Greenhouse

**Affiliations:** 1 Division of Infectious Diseases, Department of Medicine, University of California, San Francisco, San Francisco, California, United States of America; 2 Division of Experimental Medicine, Department of Medicine, University of California, San Francisco, San Francisco, California, United States of America; 3 Division of HIV/AIDS, Department of Medicine, University of California, San Francisco, San Francisco, California, United States of America; 4 Center for Biomedical Research, The Burnet Institute, Melbourne, Australia; 5 Department of Immunology and Infection, London School of Hygiene and Tropical Medicine, London, United Kingdom; 6 Infectious Disease Research Collaboration, Uganda; 7 Infectious Diseases Institute, Makerere University College of Health Sciences, Kampala, Uganda; Francis Crick Institute, UNITED KINGDOM

## Abstract

Exposure to *Plasmodium falciparum* is associated with circulating “atypical” memory B cells (atMBCs), which appear similar to dysfunctional B cells found in HIV-infected individuals. Functional analysis of atMBCs has been limited, with one report suggesting these cells are not dysfunctional but produce protective antibodies. To better understand the function of malaria-associated atMBCs, we performed global transcriptome analysis of these cells, obtained from individuals living in an area of high malaria endemicity in Uganda. Comparison of gene expression data suggested down-modulation of B cell receptor signaling and apoptosis in atMBCs compared to classical MBCs. Additionally, in contrast to previous reports, we found upregulation of Fc receptor-like 5 (FCRL5), but not FCRL4, on atMBCs. Atypical MBCs were poor spontaneous producers of antibody *ex vivo*, and higher surface expression of FCRL5 defined a distinct subset of atMBCs compromised in its ability to produce antibody upon stimulation. Moreover, higher levels of *P*. *falciparum* exposure were associated with increased frequencies of FCRL5^+^ atMBCs. Together, our findings suggest that FCLR5^+^ identifies a functionally distinct, and perhaps dysfunctional, subset of MBCs in individuals exposed to *P*. *falciparum*.

## Author Summary

A subset of “atypical” memory B cells found in individuals with high exposure to *P*. *falciparum* has been hypothesized to be dysfunctional, based on phenotypic similarities to analogous cells found in HIV-infected individuals. However, the functional capabilities of these cells have been poorly characterized in the setting of malaria exposure, and previous reports have been controversial regarding whether these cells produce antibody. In our study, we analyze the molecular programming of atypical memory B cells, find that they are dysfunctional in a manner similar to that observed in B cells from HIV-infected individuals, and present data that may reconcile previously conflicting studies. By delineating the transcriptional landscape of atMBCs and identifying expression of FCRL5 as a key marker of dysfunction, we provide a foundation for improving our understanding of the role of these cells in immunity to malaria.

## Introduction

Naturally acquired immunity is vital in reducing morbidity and mortality from *Plasmodium falciparum* malaria in endemic areas, where some individuals receive hundreds of infectious mosquito bites per year. Humoral responses to *P*. *falciparum* may be a critical component of this immunity, and *P*. *falciparum*-specific memory B cells (MBCs) are likely important in the development and maintenance of an effective response [1–3]. Unfortunately, protection from symptomatic disease takes many years to develop, during which time children living in endemic areas experience multiple episodes of symptomatic malaria, resulting in over half a million deaths annually [4–8].

One possible explanation for the slow and incomplete development of immunity to malaria is that chronic exposure to *P*. *falciparum* alters the immune response in ways that interfere with the development of protective B cell responses [9]. In particular, *P*. *falciparum* exposure has been associated with higher frequencies of circulating CD21^-^CD27^-^ “atypical” memory B cells (atMBCs) [10–17]. These cells are distinct in their surface phenotype, and possibly function, from CD21^+^CD27^+^ classical memory B cells (MBCs), which are capable of undergoing a recall response that includes proliferation and differentiation into antibody-secreting cells. The surface phenotype of atMBCs exhibits commonalities with a subset of dysfunctional B cells found in viremic HIV patients. These cells express inhibitory receptors, such as FCRL4 and SIGLEC6, that block their ability to undergo recall in response to mitogenic stimuli [18–20]. In addition to malaria and HIV, nonclassical MBC phenotypes have been identified in the context of other chronic diseases such as common variable immunodeficiency (CVID), systemic lupus erythematosus (SLE), and HCV [21–26], and they bear similarities to B cells found in the tonsils of healthy individuals [27,28]. This has led to the notion that atMBCs might represent a functionally inhibited state that results from chronic antigen exposure [11,12], in analogy to the induction of exhaustion in T cells [29,30].

Malaria-associated atMBCs were originally reported in individuals living in Mali [11], and their association with increasing exposure to *P*. *falciparum* has been corroborated in several studies using distinct cohorts from different geographical locations [10–17]. Although this association is increasingly well established, there are limited available data on the function of atMBCs in the context of malaria [11]. A recent study of atMBCs concluded that they are capable of producing *P*. *falciparum*-specific antibodies found in the serum [31], suggesting that these cells are not dysfunctional but rather may play an important role in host protection. However, this study did not define atMBCs with markers to specifically exclude antibody-producing plasmablasts, which may confound findings of antibody production. Importantly, the conclusion of antibody production was also based on indirect evidence correlating circulating antibody fragments with atMBC-encoded repertoires, which does not exclude the alternative possibility that circulating antibodies were produced by other B cell subsets. Thus, whether atMBCs are capable of producing antibody remains unclear.

A more global investigation of the functional programs expressed in malaria-associated atMBCs would help to define their role in immunity. To this end, we performed microarray-based transcriptome analysis of highly purified atMBCs from Ugandan children. Using paired comparisons to classical MBC transcriptomic profiles from the same individuals, we present a detailed examination of the functional programming of these cells. We demonstrate that atMBCs express FCRL5, but *not* FCRL4 as reported in other studies, and that expression of FCRL5 is associated with a poor capacity for antibody production. Our findings provide unique insights into the functional programming of these nonclassical MBCs and the nature of B cells in immunity to malaria.

## Results

### Transcriptional programming of atMBCs suggests decreased B cell receptor (BCR) signaling and apoptosis

**Fig 1:**
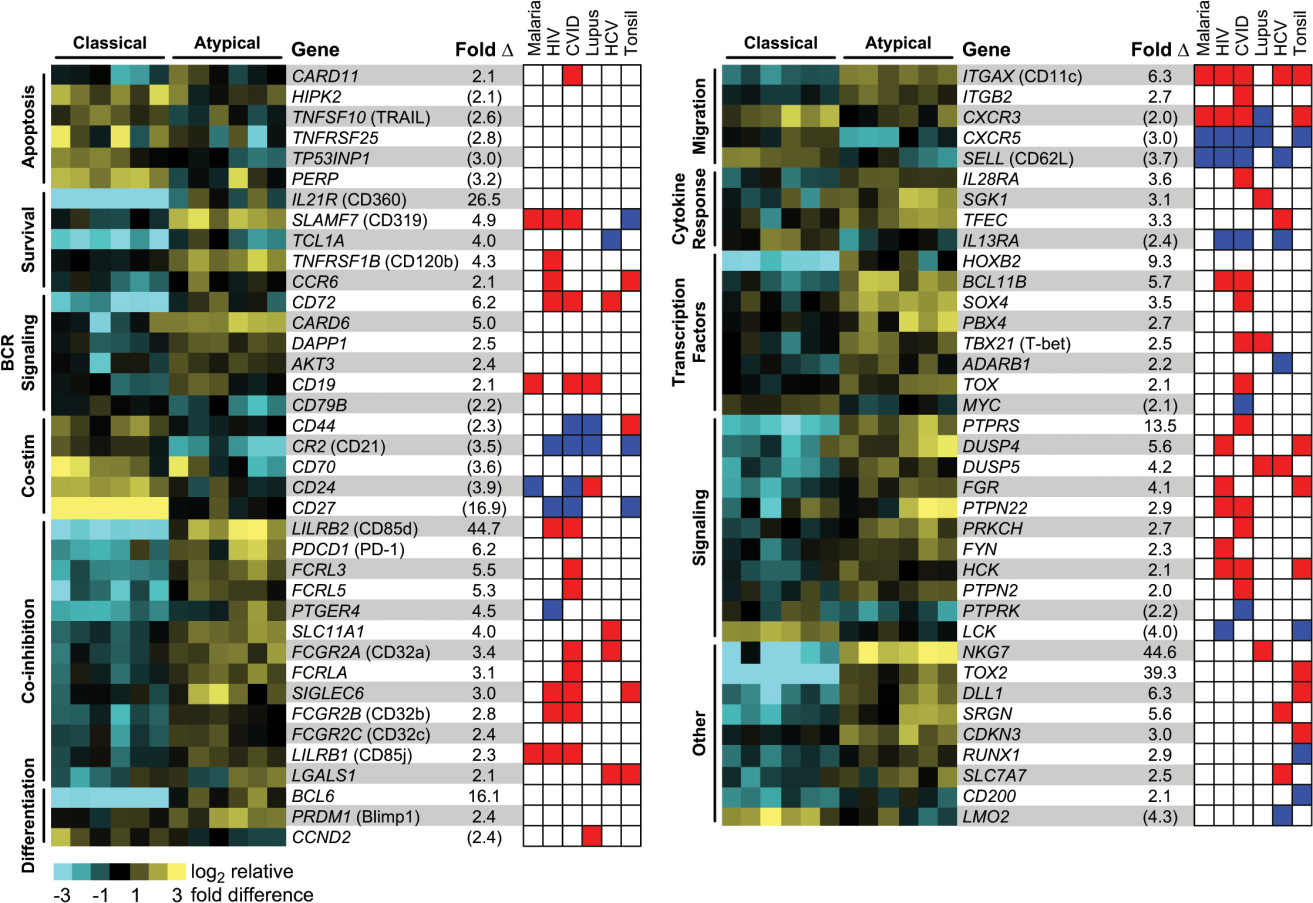
Whole-transcriptome analysis of atypical and classical MBCs from parasitemic, but asymptomatic, subjects. Heat map rows represent individual genes, and columns within each MBC grouping represent distinct individuals. Representative genes are depicted based on gene ontology associations with specific functional categories. Average fold difference in expression between atMBCs and classical MBCs pairs is shown, with values in parentheses representing lower expression in atMBCs and all other values representing higher expression in atMBCs. The red and blue heat map is a graphical depiction of the significant differential regulation of each gene in nonclassical memory B cell subsets in the context of HIV infection [18,19,45], CVID [22,23], SLE [21,24,25], HCV infection [26,46], and the tonsil [27,28], as well as previously reported expression in atMBCs in the context of malaria [11,31]. Direction of expression change was assigned based on previously published transcriptome and protein expression profiles as described in the methods, with red representing higher expression in nonclassical subsets, blue representing lower expression, and white representing the lack of any reported change.

A number of studies have established an association between higher frequencies of atMBCs and increasing exposure to *P*. *falciparum* [10–17], but the functional programming of these cells remains poorly characterized. Consistent with prior reports, we found that the frequencies of circulating atMBCs in individuals from our cohort living in a high *P*. *falciparum* transmission region in Uganda were higher than in malaria-naïve controls, and increased with age (S1 Fig). To better understand differences between atMBCs and classical MBCs, we performed microarray-based whole transcriptome comparisons of atMBCs to classical MBCs within asymptomatic parasitemic individuals living in areas of intense *P*. *falciparum* transmission. Sort-purified class-switched atMBCs (CD3^-^CD14^-^CD19^+^CD10^-^CD27^-^CD21^-^IgD^-^IgG^+^) and classical MBCs (CD3^-^CD14^-^CD19^+^CD10^-^CD27^+^CD21^+^IgD^-^IgG^+^) were processed for whole human transcriptome microarray analysis using previously described methods [32,33]. Differential gene expression analysis demonstrated that atMBCs express a transcriptional repertoire distinct from that of classical MBCs. Using a false discovery rate of 3% and a 1.5-fold change threshold, we identified 2226 differentially expressed probes representing 1479 unique genes (S1 Table). Approximately 60% of these genes were more highly expressed in atMBCs than classical MBCs. Functional enrichment analysis demonstrated significant differences in categories related to multiple B cell functions (Fig 1). For example, atMBCs exhibited lower expression of genes associated with co-stimulation of BCR signaling, such as *CD79b*, *CD70*, *CD24*, and *CD44*. This was accompanied by higher expression of regulators of BCR signaling (*LILRB2*, *ITGAX*), Fc receptor family inhibitory receptors (*FCRLA*, *FCRL3*, *FCRL5*), and genes known to promote B cell anergy and exhaustion (*SIGLEC6*, *PDCD1*, *LGALS1*). Together, the differences in regulation of these genes are suggestive of cell-intrinsic down-modulation of BCR signaling in atMBCs.

Genes involved in apoptosis, particularly those related to p53 signaling, were expressed at lower levels in atMBCs than classical MBCs. For example, *HIPK2*, a pro-apoptotic protein that phosphorylates p53 in response to DNA damage [34–36], exhibited lower expression in atMBCs. Other pro-apoptotic genes with lower relative expression in atMBCs included *TP53INP1*, which promotes cell cycle arrest and apoptosis [37]; *TNFSF10* (TRAIL), a gene target in the p53 cell death pathway [38]; *PERP*, a mediator of p53-dependent apoptosis [39]; and *TNFRSF25* (Death Receptor 3), which functions similarly to CD95 (Fas), with over-expression leading to NF-κB induction and apoptosis [40]. We concomitantly detected higher expression of *TNFRSF1B* and *IL21R*, both of which can promote B cell survival [41–44]. Together, suppressed expression of these pro-apoptotic factors could promote the survival of atMBCs, suggesting one mechanism by which they might accumulate with increasing parasite exposure.

To better understand the relationship of atMBCs to nonclassical memory B cell subsets found in other disease contexts, we collated data from diverse studies characterizing the mRNA and protein expression levels of signature genes in these cells [11,18,20–28,31,45,46] (Fig 1). The direction of gene expression in malaria-associated atMBCs relative to classical MBCs corresponded well with gene expression patterns of other nonclassical memory B cell subsets; specifically, 88% of the changes occurred in the same direction, with the highest proportion of overlap occurring with CD27^-^CD21^-^ cells in HIV (89%, 21 of 23 genes) and CD21^lo^ cells in CVID (97%, 32 of 33 genes). Functional overlap extended to most categories, with the notable exception of apoptosis. Together, these data suggest that in addition to similarity in surface phenotypes, atMBCs may exhibit functional similarity to nonclassical memory B cells associated with other chronic diseases.

Notably, we detected a decrease in expression of *CXCR3* in atMBCs, despite reports that this marker is increased on malaria-associated atMBCs and similar cells in the tonsil and in individuals with HIV, SLE, and CVID [11,20–22,27,45]. We did not detect a relative increase in expression of *FAS* (CD95), though this has been reported for cells in the tonsil and in individuals with HIV, SLE, and CVID [21–23,28,45]. Other genes previously described to be differentially expressed in similar B cells from other contexts, but not detected in our microarray analysis, included *LAIR1*, *CXCR4*, and the genes encoding caspase-1 (*CASP1*) and caspase-9 (*CASP9*), which further distinguishes malaria-associated atMBCs with reports from HIV, SLE, and CVID [18,22,23,25,26,28]. Thus, although there are abundant commonalities between malaria-associated atMBCs and cells of similar surface phenotype associated with other diseases, there are also unique aspects that differentiate malaria-associated atMBCs from other exhausted and nonclassical memory B cell subsets (S2 Table).

### Heterogeneity in surface phenotype and function of CD21^-^CD27^-^IgG^+^ B cells

**Fig 2:**
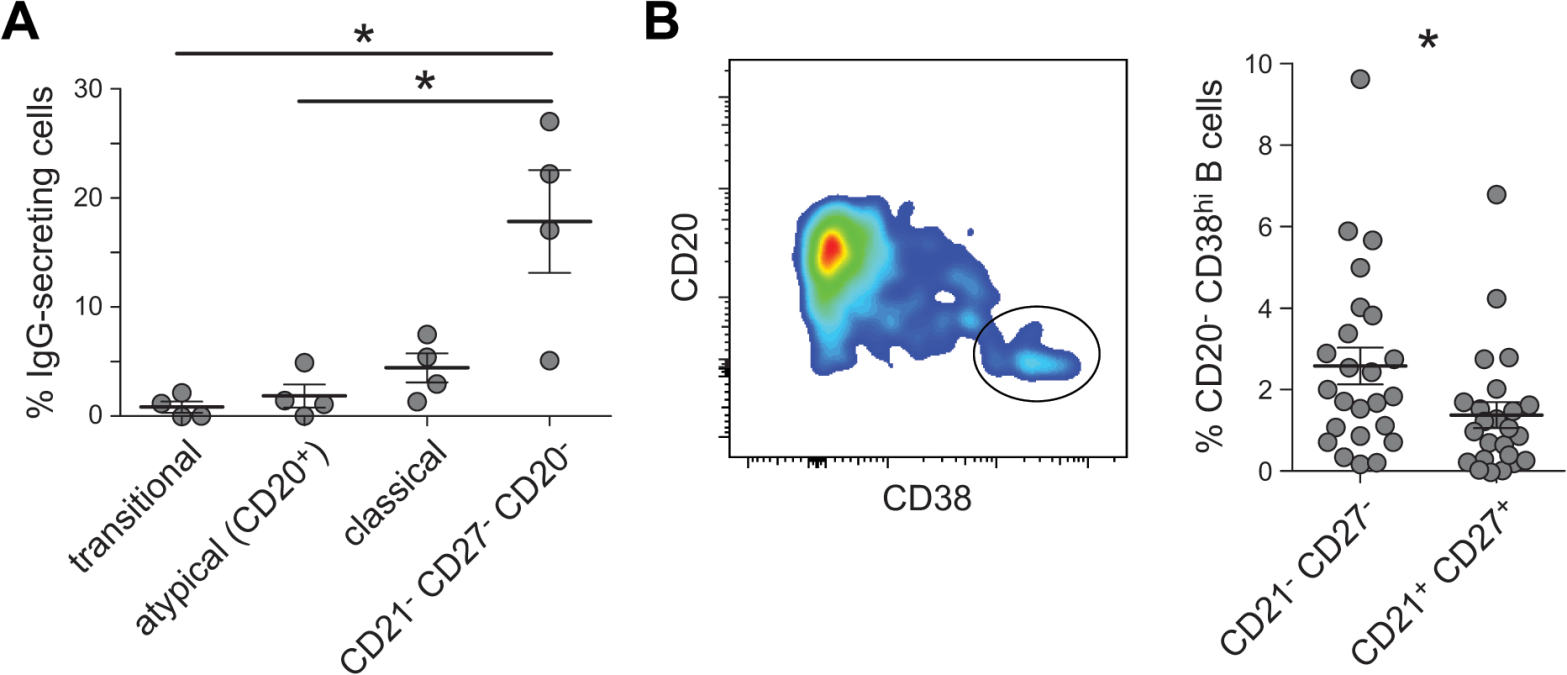
Spontaneous IgG secretion by different B cell subsets. (**A**) Sorted transitional cells (CD19^+^CD10^+^), CD20^+^ atMBCs (IgG^+^CD21^-^CD27^-^CD19^+^), classical MBCs (IgG^+^CD21^+^CD27^+^CD19^+^), and CD27^-^ plasmablasts (CD20^-^IgG^+^CD21^-^CD27^-^CD19^+^) were cultured on anti-IgG ELISpot plates for 18 h without additional stimulation. (**B**) Gating strategy and frequencies of CD38^hi^ cells in the above plasmablast gating strategy.

A key functional phenotype of exhausted MBCs found in HIV-viremic individuals is their decreased ability to differentiate into antibody-secreting cells [19,20], leading early reports to propose that malaria-associated atMBCs might be similarly dysfunctional [11]. Consistent with this, we observed that atMBCs expressed higher levels of *SIGLEC6* and *BCL6*, which negatively regulate B cell proliferation and differentiation [19,47,48]. Similarly, *PDCD1*, which encodes the signaling regulator PD-1 [49], was more highly expressed in atMBCs than in classical MBCs. Surprisingly, we also observed that atMBCs express higher levels of *PRDM1* (the gene encoding BLIMP-1), a regulator of plasmablast differentiation which acts in opposition to BCL6. This raised the possibility that plasmablasts comprised a subset of these CD21^-^CD27^-^IgG^**+**^ cells, a phenotype previously used to define atMBCs [11,31]. To test this hypothesis, we examined spontaneous antibody production from CD20^+^ and CD20^-^ subsets in the absence of stimulation, which is a property of antibody-secreting cells such as CD20^-^ plasmablasts. We found that among CD20^+^ atMBCs (CD19^+^IgG^+^CD10^-^CD27^-^CD21^-^CD20^+^), only 1.6% of cells spontaneously secreted IgG *ex vivo* (Fig 2A). In contrast, 18% of cells with a similar surface phenotype but lacking expression of CD20 spontaneously secreted IgG. These CD19^+^IgG^+^CD10^-^CD27^-^CD21^-^CD20^-^ cells also expressed high levels of CD38, which is consistent with the surface phenotype of plasmablasts/plasma cells (Fig 2B). We found that on average, 2.6% of the cells within the CD19^+^IgG^+^CD10^-^CD27^-^CD21^-^ gate were CD20^-^ and CD38^hi^. Therefore, to distinguish atMBC from this minor population of likely plasmablasts, we incorporated CD20 and CD38 into all analyses below, defining atMBCs as CD19^+^ CD20^+^ CD21^-^ CD27^-^ CD38^int/lo^ IgG^+^. Among the genes we identified as relatively enriched in atMBCs, only 6 (0.5%) were identified as being enriched in plasmablasts in a previous study [50]. Thus, the likely inclusion of a small number of plasmablasts along with atMBCs was unlikely to have significantly affected our microarray results.

### Differential surface phenotypes of classical and atypical MBCs

The surface phenotype of atMBCs is most commonly defined by the absence of expression of CD21 and CD27. In accord with protein levels, transcripts of both *CR2* (the gene encoding CD21) and *CD27* were significantly lower in atMBCs than classical MBCs, indicating that down-regulation of the expression of these markers occurs, at least in part, at the level of transcription. Previous studies also described differential expression of protein levels of CD85j, CD11c, CXCR5, CD24, CD84, and CD319 [11,13,31], which we corroborated at the transcript level as differential expression of *LILRB1*, *ITGAX*, *CXCR5*, *CD24*, *CD84*, and *SLAMF7*, respectively (Fig 1). Notably, these markers represent high confidence signatures, given that they have been identified as markers of atMBCs at both the mRNA and protein levels in studies of distinct cohorts performed by different laboratories. In addition to the above, we detected significantly increased expression of *LILRB2* (CD85d), *TNFRSF1B* (CD120b), and *IL21R* (CD360) in atMBCs relative to classical MBCs. Expression of *LILRB2* and *TNFRSF1B* was previously reported to be increased in exhausted B cells during HIV infection [18], and *LILRB2* and its encoded protein, CD85d, were expressed in CD21^lo^ B cells from patients with combined variable immunodeficiency (CVID) [23].

**Fig 3:**
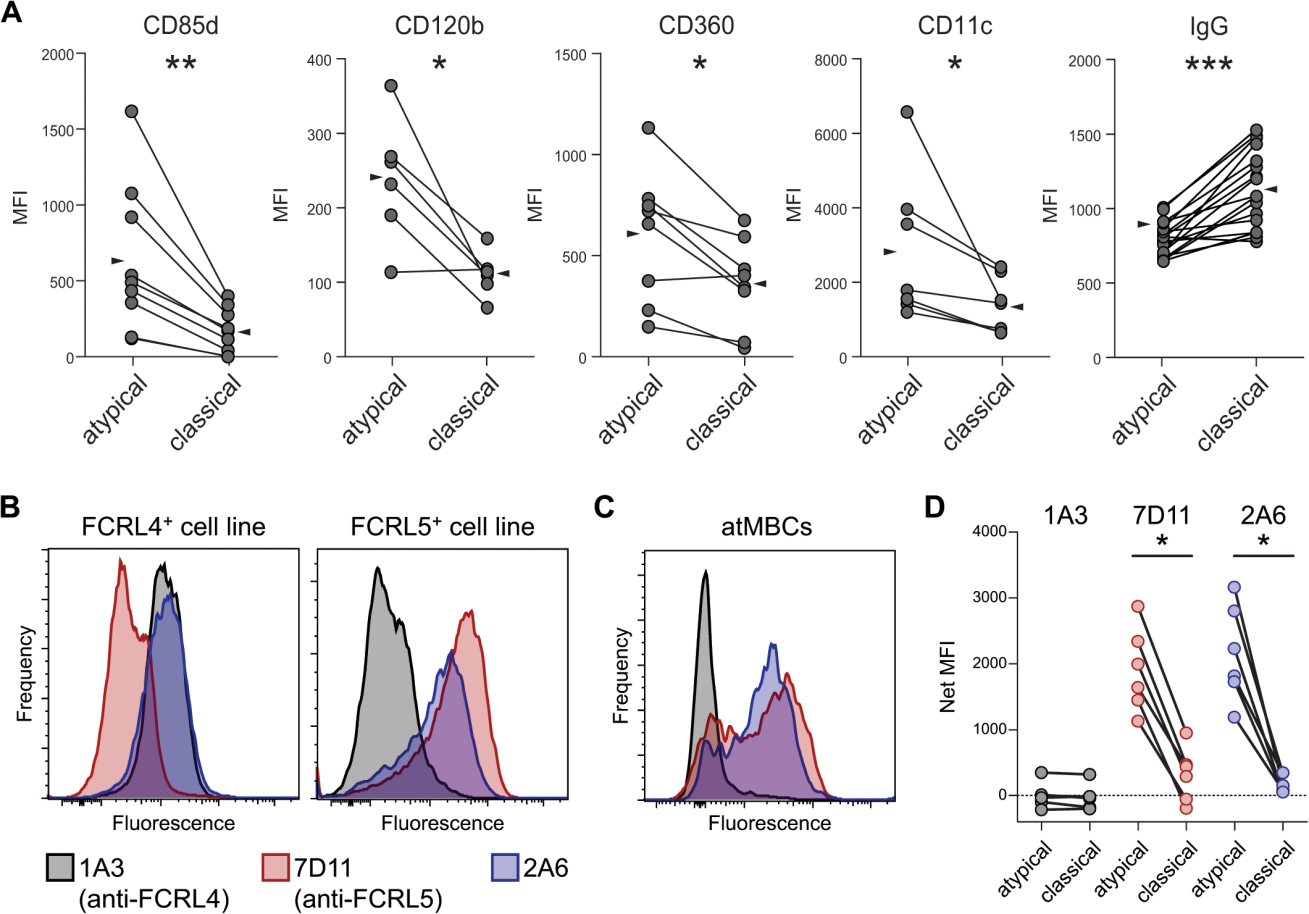
Phenotypic characterization of surface proteins on IgG^+^ atypical MBCs. (**A**) Surface expression, expressed as median fluorescence intensity (MFI), of CD85d, CD120b, CD360, CD11c, and IgG (BCR) on IgG^+^ atMBCs and IgG^+^ classical MBCs. Lines between symbols denote MBC subsets from the same subject. Wedges represent means. (**B**) Labeling of SVT2 mouse fibroblast cell lines that express full-length human *FCRL4* or *FCRL5* protein by monoclonal antibodies 2A6, 1A3, and 7D11. (**C**) Labeling of human atMBCs with monoclonal antibodies 2A6, 1A3, and 7D11. (**D**) Isotype-subtracted MFI of FCRL family member expression (“Net MFI”) on atypical and classical MBCs from highly *P*. *falciparum*-exposed individuals. Statistical significance was determined using the Wilcoxon signed-rank test. *, p < 0.05; **, p < 0.01

We corroborated the expression of CD85d, CD120b, and CD360 at the protein level on samples from our highly *P*. *falciparum-*exposed individuals by surface staining of atMBCs (Fig 3A). As in previous studies, we also found CD11c protein to be significantly increased on the surface of atMBCs relative to classical MBCs. The Ig-beta chain of the BCR, encoded by *CD79B*, is required for proper trafficking of the BCR; diminished expression of *CD79B* in atMBCs would be predicted to result in lower levels of surface-localized BCR. As previously reported by others [31,51], we found surface IgG levels to be significantly lower on atMBCs than classical MBCs from the same individuals (Fig 3A), consistent with down-modulation of surface BCR.

### FCRL5, but not FCRL4, is expressed by a subset of atMBCs

FCRL4 protein was previously reported to be expressed on malaria-associated atMBCs [13,31], and elevated gene and/or protein expression has been reported for HIV-associated exhausted MBCs [18,20], tonsillar B cells [27,28], and nonclassical memory B cells associated with CVID and hepatitis C infection [22,26,46]. Surprisingly, we did not detect significantly increased expression of *FCRL4* by atMBCs in our microarray analysis. Quantitative RT-PCR analysis of *FCRL3*, *FCRL4*, and *FCRL5* corroborated the microarray data, demonstrating that *FCRL3* and *FCRL5*, but not *FCRL4*, transcripts were present at higher levels in atMBCs than classical MBCs (S2 Fig). FCRL3 and FCRL5 share 28–60% extracellular amino acid sequence identity with FCRL4 [52], suggesting that antibodies used to detect surface-localized FCRL4 in other studies might have cross-reacted with other FCRL family members. To test this possibility, we assessed the specificity of various anti-FCRL antibodies using cell lines constitutively expressing FCRL4 or FCRL5 [53]. Consistent with the original study that produced these antibodies [53], the anti-FCRL4 antibody clone 1A3 and the anti-FCRL5 antibody clone 7D11 bound specifically to the expected cell lines (Fig 3B). In contrast, the widely used anti-FCRL4 antibody clone 2A6 [27], which was employed in previous malaria-associated atMBCs studies [11,31], bound strongly to both FCRL4- and FCRL5-expressing cell lines. Thus, the 2A6 antibody binds to both FCRL4 and FCRL5, whereas the 1A3 and 7D11 antibodies are specific for FCRL4 and FCRL5, respectively.

Having determined the specificity of these antibodies, we measured the surface expression of FCRL4 and FCRL5 on MBCs from 8–10 year old children and adults from our high exposure Ugandan cohort; all selected subjects were smear positive for *P*. *falciparum* but lacked fever. Consistent with previous reports [11,31], the nonspecific 2A6 clone labeled atMBCs more strongly than classical MBCs (Fig 3C). Similar results were seen with the anti-FCRL5 antibody 7D11. In contrast, the anti-FCRL4 antibody 1A3 failed to exhibit binding above an isotype control background to either atMBCs or classical MBCs. Given that these protein level data are consistent with our microarray and qRT-PCR observations that *FCRL5*, but not *FCRL4*, is more highly expressed by malaria-associated atMBCs than classical MBCs, it is likely that FCRL5 is the actual target recognized on these cells by previous malaria studies that used clone 2A6 [11,31].

### FCRL5 defines a subset of atMBCs with a distinct surface phenotype

**Fig 4:**
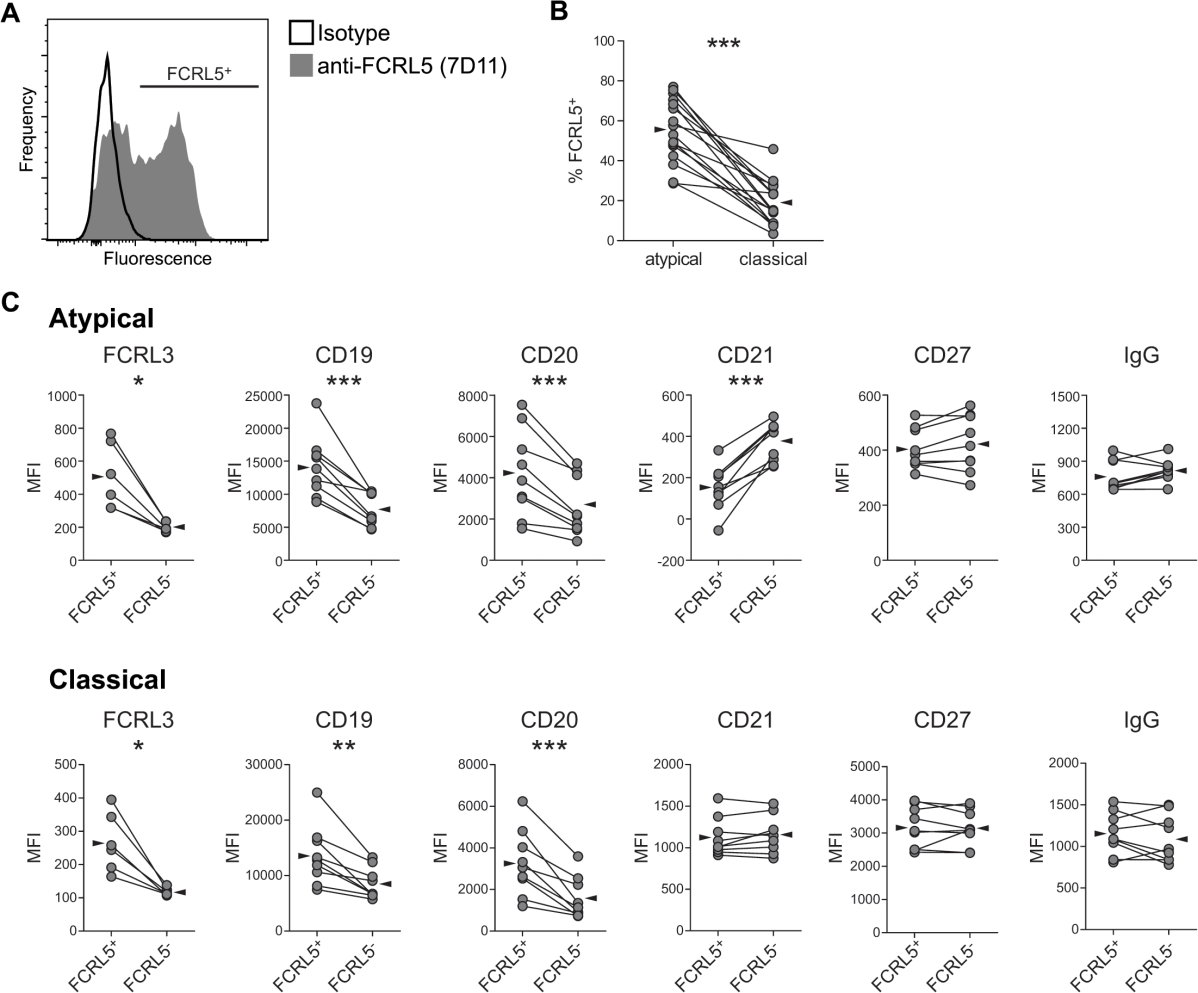
FCRL5 expression defines a phenotypically distinct subset of IgG^+^ atMBCs. (**A**) Representative plot showing heterogeneous expression of FCRL5 on IgG^+^ atypical MBCs. Individual FCRL5^+^ atypical and classical MBC frequencies were determined using gates set with a “fluorescence minus one” control with IgG2b isotype control antibody. (**B**) Proportion of atypical and classical IgG^+^ MBCs expressing FCRL5. Reported frequencies have been subtracted for isotype-labeled background. (**C**) Median fluorescence intensity (MFI) of surface markers on FCRL5^+^ vs. FCRL5^-^ atMBCs and FCRL5^+^ vs. FCRL5^-^ classical MBCs. Statistical significance was determined using the Wilcoxon signed-rank test. *, p < 0.05; **, p < 0.01; ***, p < 0.001.

FCRL5 expression followed a heterogeneous distribution on atMBCs (Fig 4A). The proportion of atMBCs that were FCRL5^+^ between individuals was variable (mean 53%, range 18–74%), but was consistently higher than the proportion of classical MBCs that were FCRL5^+^ in the same individual (mean 23%, range 10–52%; p < 0.001) (Fig 4B). Given the non-uniformity in FCRL5 expression on atMBCs, we considered the possibility that FCRL5^+^ atMBCs might represent a distinct subset from FCRL5^-^ atMBCs. To assess this possibility, we compared the surface phenotypes of FCRL5^-^ and FCRL5^+^ atMBCs and classical MBCs. Compared to FCRL5^-^ atMBCs, the FCRL5^+^ subset expressed significantly higher levels of FCRL3, CD19, and CD20, but lower levels of CD21, with no significant difference in either CD27 or IgG expression (Fig 4C). Similar trends were observed for classical MBCs, with the exception that CD21 was unchanged. These findings are consistent with FCRL5^-^ and FCRL5^+^ cells being distinct, though perhaps developmentally and/or functionally related, subsets of memory B cells.

### FCRL5^+^ atMBCs increase with *P*. *falciparum* exposure

**Fig 5:**
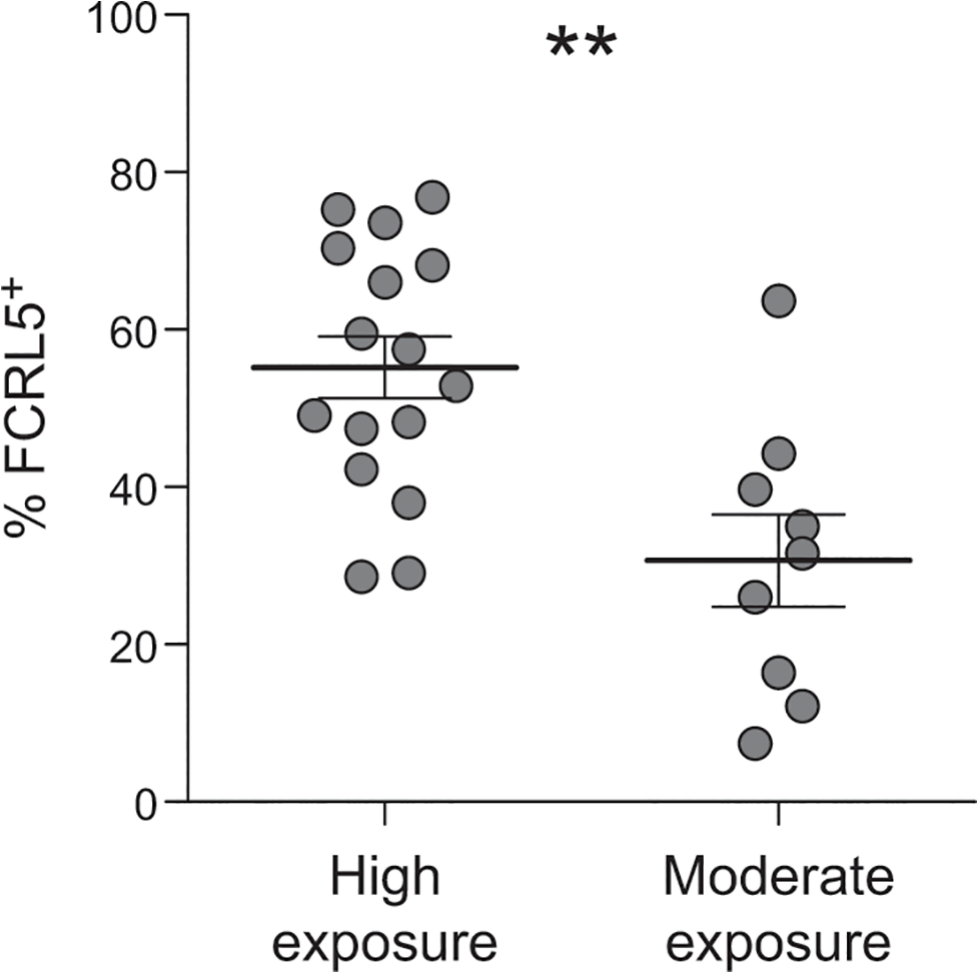
Higher exposure to *P*. *falciparum* is associated with a higher proportion of atMBCs that express FCRL5. The proportion of FCRL5^+^ atypical MBCs from individuals living in high exposure (n = 16; Nagongera, Uganda; annual entomologic inoculation rate = 310) vs. moderate exposure (n = 9; Walukuba, Uganda; annual entomologic inoculation rate = 2.8) is shown, p = 0.004. Statistical significance was determined using the Wilcoxon rank-sum test. Multivariate linear regression, including age of subject, yielded similar results.

A number of studies have reported that the frequency of atMBCs increases with age and *P*. *falciparum* exposure [11–13,15,16]. If exposure induces phenotypic changes in atMBCs consistent with reduced responsiveness, we predicted that the FCRL5^+^ subpopulation of atMBCs would similarly increase with *P*. *falciparum* transmission intensity. To test this hypothesis, we compared expression of FCRL5 on atMBCs from study participants living in Nagongera, Uganda, where transmission is very high, to those from Walukuba, a periurban area of Uganda where transmission is ~30 fold lower [54,55]. Subjects living in the area of higher malaria transmission had a significantly higher proportion of FCRL5^+^ atMBCs than subjects living in the area with lower transmission (Fig 5; mean difference of 25%, p = 0.004 by Wilcoxon rank-sum test and in multivariate regression including age).

### FCRL5^+^ classical and atypical MBCs exhibit inhibition of antibody production upon stimulation

**Fig 6:**
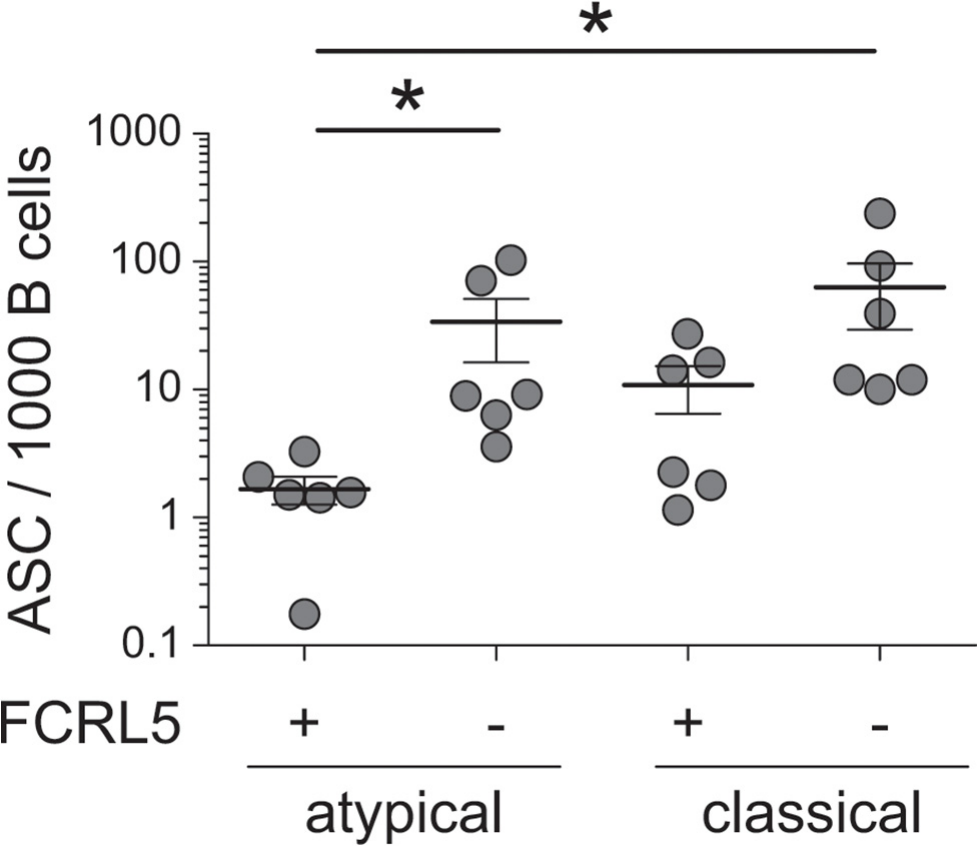
Recall antibody secretion by different B cell subsets. Sorted FCRL5^+^ and FCRL5^-^, atypical (CD20^+^CD21^-^CD27^-^IgG^+^) and classical (CD20^+^CD21^+^CD27^+^IgG^+^) MBCs were stimulated for 4 days with CpG, F(ab’)_2_ anti-IgG, and autologous T cells. IgG-secreting cells were detected by IgG ELISpot and are reported as the number of IgG secreting cells per 1000 cells sorted on day 0. ASC, antibody-secreting cells. Statistical significance was determined using the Wilcoxon signed-rank test. *, p < 0.05.

Having shown that CD20^+^ atMBCs are poor spontaneous producers of antibody *ex vivo*, we evaluated their capacity to differentiate into antibody-secreting cells following stimulation (*i*.*e*., recall). Given the heterogeneity in FCRL5 expression in atMBCs and the potential inhibitory role of this surface receptor [56], we also evaluated whether this surface marker distinguished subsets with different capacities to undergo recall. FCRL5^-^ and FCRL5^+^ subsets of atMBCs and classical MBCs were isolated by flow cytometry and stimulated for 4 d *in vitro* with an activating anti-BCR antibody and CpG to induce a recall response. Following stimulation, FCRL5^-^ classical MBCs exhibited robust production of antibody as expected, with a mean of 6.3% of these cells capable of secreting IgG (Fig 6). In comparison, FCRL5^-^ atMBCs exhibited reduced capacity to produce antibody (3.4% IgG-secreting cells), though this difference did not reach statistical significance. More strikingly, FCRL5 expression defined strongly inhibited subsets of both classical and atMBCs, with only 1.1% of FCRL5^+^ classical MBCs and 0.2% of FCRL5^+^ atMBCs capable of a recall response. Of note, FCRL5^-^ atMBCs produced a higher proportion of IgG-secreting cells than FCRL5^+^ classical MBCs. Thus, expression of FCRL5, more so than the traditional subset-defining markers, strongly delineates functionally distinct groups of memory B cells and is correlated with inhibition of antibody production.

## Discussion

We have performed a detailed molecular characterization of malaria-associated atMBCs, beginning with an unbiased transcriptome-wide comparison with classical MBCs and leading to functional characterization of atMBC subsets defined by differential expression of FCRL5. We show that in comparison to classical MBCs, atMBCs obtained from individuals living in an area of intense malaria transmission in Uganda have a distinct transcriptional program, with down-modulated BCR signaling that may contribute to reduced function, and changed apoptosis programs which may contribute to accumulation. This analysis reveals new surface markers that identify atMBCs, particularly FCRL5, which we show is a specific correlate of poor recall capacity.

Based on the surprising finding that *FCRL5*, but not *FCRL4*, was enriched for expression in atMBCs, we confirmed that an anti-FCRL4 antibody used in many prior studies cross-reacts with FCRL5. The extent to which these molecules have been confused in the literature is unclear, and it is certainly possible that FCRL4 is expressed by some analogous B cell subsets given that increased gene expression and functional studies of *FCRL4* perturbation have been reported [19,22,28,46,57]. A re-examination of antibody specificity is warranted to determine if in some studies, the functional consequences of FCRL5 expression might have been missed and/or ascribed to FCRL4 as a result of non-specific recognition or perturbation. Interestingly, some evidence suggests that FCRL5 is a receptor for IgG, which circulates at high levels during malaria [58,59]. Thus, FCRL5 expression on B cells could participate in a feedback mechanism for IgG homeostasis during hypergammaglobulinemia, thereby impacting memory B cell responses.

In accord with studies of an analogous subset in HIV-viremic individuals [19,20], we find that atMBCs in malaria-exposed individuals are comparatively ineffective at producing antibody *ex vivo*, either spontaneously or following re-stimulation. These findings contrast with those of a recent study which concluded that atMBCs actively produce protective antibodies *in vivo* [31]. However, the authors reached this conclusion based on the indirect observations that transcripts of secretory IgG, along with membrane IgG, were detected in atMBCs, and that BCR sequences from some atMBCs matched those of serum IgG fragments in a single subject. In light of our findings, two alternative possibilities that could explain the detection of secretory transcripts are that: a) these transcripts were derived from a minority population of CD20^-^ plasmablasts and not atMBCs; or b) transcripts were derived from CD20^+^ atMBCs but these cells were not actively producing antibody, possibly due to transient or permanent arrest of differentiation by inhibitory molecules such as FCRL5. To additionally explain the detection of overlapping repertoires in serum IgG and atMBCs [31], we suggest the possibilities that: a) atMBCs do not themselves produce antibody, but at some frequency can eventually differentiate into antibody secreting cells; or b) atMBCs do not produce antibody nor do they differentiate into antibody secreting cells, but antibody secreting cells and atMBCs share antibody repertoires [60] due to derivation from a common progenitor such as classical MBCs. In any case, CD20^+^ atMBCs have a relative decrease in the capacity to secrete antibody in response to stimulation versus classical MBCs. However, this difference is modest compared with the marked decrease seen in the FCRL5^+^ subsets of either MBC population. Based on the magnitude of the effect, the traditional subset markers that distinguish atMBCs from classical MBCs (CD21 and CD27) are less effective than FCRL5 in defining a functionally distinct subset. This raises the question of how best to consider the relationships of the various sub-populations, and suggests the possibility that up-regulation of FCRL5 expression precedes down-regulation of CD21 and/or CD27, in a progression through which MBCs adopt a state of reduced antibody production. This model is also in accord with a very recent report that BCR variable region sequences in atMBCs are largely indistinguishable from those found in classical MBCs [60]. Further experimentation to better define the relationships between these subsets is urgently needed, as this will have an important influence on our thinking about the ontogeny and function of these populations.

Consistent with down-modulation of B cell functions, increasing evidence suggests that higher levels of exposure to *P*. *falciparum* induce immunoregulatory processes that dampen infection-associated immune activation [10,32,61]. This development of immunological tolerance might underlie the decreasing severity of malaria disease with increasing exposure and age, but may come at the expense of inhibiting sterilizing immunity. Similar to upregulation of expression of immunoregulatory receptors on γδ T cells [32], we show here that FCRL5 expression on B cells is associated with higher levels of exposure to *P*. *falciparum*. In turn, FCRL5 is associated with poor antibody production, suggesting that upregulation of this receptor may be a mechanism of cell-intrinsic immunoregulation. We note, however, that atMBCs are associated with increasing age and exposure to *P*. *falciparum*, the same factors which are associated with acquired immunity [11–13,15,16,62]. Acquired immunity allows individuals, like those studied here, to remain asymptomatic while parasitemic, not to receive antimalarial therapy, and therefore to remain infected. It is possible that immune activation associated with this state of asymptomatic parasitemia in part drives the accumulation of atMBCs and affects aspects of their phenotype, such as the expression of FCRL5. Frequencies of atMBC appear to decrease following elimination of *P*. *falciparum* exposure [10,16], but further studies will be required to assess the dynamics of atMBC frequency and phenotype in response to acute and chronic *P*. *falciparum* infection. It remains to be determined whether atMBCs are truly dysfunctional, with immunity being acquired despite their accumulation; play an immunoregulatory role, aiding in the development of tolerance to *P*. *falciparum* infection; or have an as yet undefined role in anti-parasite immunity, *e*.*g*., antigen presentation. Further functional studies will also be required to elucidate the roles of FCRL5 and other similarly expressed immunoregulatory molecules in this process.

Given the similarities between atMBCs and similar B cell subsets found in other contexts of chronic antigen exposure, such as HIV infection, HCV infection, SLE, and CVID [11,18–26,31,45,46], it may be that these cells are not so “atypical” at all. These subsets all share a similar biomarker phenotype (CD19^+^CD21^lo/-^CD27^-^) and are all hypothesized or demonstrated to have refractory responses to B cell mitogens. In addition to functional and biomarker similarities, we found that many of their gene expression signatures were also shared, including similarities in expression of immunoregulatory receptors, proteins involved in migration, and BCR co-stimulatory transcripts, which were down-regulated. However, key differences from other studies were also observed, especially with regard to B cell trafficking and survival [11,19,21–23,27,45]. It is possible that the differential expression of these markers is rooted in ontogeny; however, these markers could also reflect contextual differences, such as those driven by tissue localization, kinetics, or differences in the antigenic and/or inflammatory environment. Further studies will be needed to better define the relationships of these populations to one another through detailed functional and global transcriptomic analyses.

In summary, comparison of the gene expression of malaria-associated atMBCs vs. classical MBCs highlights key differences in these subsets and provides a foundation for comparison with analogous subsets seen in other conditions of chronic antigen exposure. High expression of FCRL5 defines distinct subsets of MBCs and appears to be a key marker of functional deficiency, at least with respect to the ability to secrete antibody in response to stimulation. Further studies of the function of these cells will be required to define their relevance to disease and immunity.

## Materials and Methods

### Ethics statement

Ethical approval was obtained from the Makerere University School of Medicine Research and Ethics Committee, the Uganda National Council for Science and Technology, the London School of Hygiene & Tropical Medicine Ethics Committee, and the University of California, San Francisco Committee on Human Research. All adult study participants provided written informed consent, and a parent or guardian of all child participants provided written informed consent on their behalf.

### Study population

Samples were obtained from participants enrolled in cohort studies as part of the East African International Center for Excellence in Malaria Research in Uganda. These cohorts of children aged 6 months—10 years of age and their adult primary caregivers were followed for all their health care needs in dedicated study clinics as previously described [55]. Samples for the majority of experiments came from parasitemic, but non-symptomatic, children 8–10 years old and adult caregivers from the Nagongera cohort in Tororo District, where malaria transmission is very high (annual entomological inoculation rate ~ 310 infectious bites per person per year) [54,55]. To compare phenotypes in different malaria transmission settings, samples were also analyzed from the Walukuba cohort in Jinja District where transmission is lower (annual entomological inoculation rate ~ 2.8 infectious bites per person per year). Older children and adults were selected since individuals in these age ranges have previously been shown to have the highest frequencies of atMBCs [11,13]. Subjects without fever were selected to avoid transient effects on B cell function associated with inflammation from symptomatic malaria or other acute illness. Subjects with documented parasitemia by microscopy were selected to keep subjects as similar to each other as possible; asymptomatic parasitemia is common in older children and adults in high transmission settings and those without documented parasitemia may or may not have had submicroscopic parasitemia.

### Microarrays

For transcriptomic analysis of atMBCs, samples were selected from children aged 8–10. Approximately ten million cryopreserved PBMCs from each child were stained with antibodies specific for CD3 (clone UCHT1), CD14 (clone M5E2), CD19 (clone HIB19), CD10 (clone HI10a), CD38 (clone HIT2), CD27 (clone O323), CD21 (clone B-ly4), IgG (clone G18-145) (all BioLegend); and IgD (clone IA6-2) (BD Biosciences) (see S3 Fig for gating strategy). Classical and atMBCs were processed for microarray analysis as previously described [32,33]. In brief, cell subsets were isolated to >99.8% purity using two successive rounds of purity-optimized sorting on a FACSAria, with 5,000 total cells on the second round sorted directly into 100 μl RNAqueous Micro lysis buffer. RNA was isolated with the RNAqueous Micro kit (Life Technologies), and was amplified in two rounds with the Amino Allyl MessageAmp II kit (Life Technologies). Amplified RNA was covalently labeled with Cy3 and hybridized to SurePrint G3 Unrestricted GE 8x60K human V2 gene expression microarrays (Agilent Technologies). Microarrays were scanned on an Agilent microarray scanner at 3 μm resolution into a 20-bit TIFF, and raw intensities were extracted with Agilent Feature Extraction. Raw intensities were log_2_-transformed and quantile-normalized using the R package limma [63]. Probes not expressed above background (normalized intensity of 128) in either sample group were removed from the data set. Significantly differentially expressed genes were identified using Significance Analysis for Microarrays in a paired comparison using a false discovery rate of 3% and 1.5-fold change threshold [64], and expression values were median centered across samples for visualization as heat maps. Functional enrichment analysis was performed using DAVID [65], using a Benjamini-corrected p value of 0.05 to determine significance. All microarray data are available in the NCBI Gene Expression Omnibus under accession number GSE64493.

### Comparison to published studies of nonclassical B cell subset gene and protein expression

We evaluated data from 14 studies that reported transcriptional or protein differences in nonclassical MBCs compared to controls in the contexts of malaria, HIV, CVID, SLE, and HCV, as well as data for tonsillar B cells, where FCRL4^+^ B cells were first described [11,19,21–28,31,45]. Studies for comparison were identified by searching PubMed for “FCRL4 B cells” or “FCRL5 B cells” [11,18,20,22,23,31]. We then searched on the diseases identified from “FCRL4 B cells” and “FCRL5 B cells” search terms to include studies with relevant transcript and protein information in B cells that did not specifically identify FCRL4 or FCRL5 [21,25,45]. Nonclassical MBCs were defined differently between studies as follows: CD21^lo/-^CD27^-^ in the context of *P*. *falciparum* exposure, HIV, CVID, and HCV cirrhosis [11,20,22,23,31,46]; HIV-specific CD21^lo/-^CD27^-^ [18]; IgD^-^CD27^-^ in SLE [21,24]; CD21^lo^CD27^+^ in HCV with mixed cryoglobulinemia [26]; FCRL4^+^ (CD21^lo/-^CD27^-^) in the tonsil [27,28]; and bulk B cells from subjects with HIV or SLE [25,45]. Transcriptional or protein differences in nonclassical MBCs were measured in comparison to either classical MBCs (CD21^+^CD27^+^) [11,31,46], HIV-specific classical MBCs [18], activated/classical MBCs (CD27^+^CD21^+/-^) [20,21], CD27^+^IgD^+/-^ B cells [24], FCRL4^-^ B cells in the tonsil [27,28], CD21^lo^CD27^+^ cells in HCV without mixed cryoglobulinemia [26], or bulk B cells from healthy donors [22,25,45]. Our comparison includes genes and proteins that were determined to be significantly differentially regulated in at least one of the studies above as well as in our own analysis, and reports the direction of the change.

### qPCR analysis

Reverse transcription was performed on 600 ng aminoallyl-incorporated amplified RNA using SuperScript III Reverse Transcriptase (Life Technologies) and poly dT_20_V oligonucleotide primer in a 20 μl reaction. Samples were incubated with primer for 10 min at 70°C prior to addition of RT to allow primers to anneal. After addition of RT, tubes were incubated for 10 min at 25°C, then 50 min at 42°C, then 15 min at 70°C to inactivate RT, following previously published methods [66]. One μl RNase H was added and samples were incubated at 37°C for 20 min to degrade the input RNA. Samples were diluted 1:5 in nuclease-free water and 5 μl of diluted sample was used in a 25 μl quantitative PCR reaction using PerfecTa 2x qPCR Mix (Quanta). Primers used in the qPCR reactions were biased toward the 3' end of mRNA transcripts, and annealed no further than 500 bp upstream from the polyA tail, to account for product shortening during amplification. Specific sequences used in this study were *ACTB-F*: 5’-AGTTCACAATGTGGCCGAGGA-3’; *ACTB-R*: 5’-TGTGTGGACTTGGGAGAGGA-3’; *FCRL3-F*: 5’-GAGGGCCCTCAGCTCCTA-3’; *FCRL3-R*: 5’-AAAGGGAAACAAAATATTTGGAGCA-3’; *FCRL4-F*: 5’-AAAACTTAAGTACCAACTCTCCAAA-3’; *FCRL4-R*: 5’-AATAAAACCTCTCTGCAAGGAGT-3’; *FCRL5-F*: 5’-AGAACAAACTCCACCCTAATGTG-3’; and *FCRL5-R*: 5’-CCAAGAAGAGCCATTTTTCAGTTTG-3’. *FCRL* transcript levels were normalized to levels of actin mRNA.

### Flow cytometry

Samples were selected from children and adults over 8 years old, unless specifically noted otherwise. All had concurrent asymptomatic parasitemia as identified by microscopy (blood smear positive, in the absence of fever). B cell subsets were defined using the antibodies described above, with the addition of CD20 (clone B9E9) (Beckman Coulter), as follows, unless otherwise specified: atypical MBCs (CD19^+^CD20^+^CD21^-^CD27^-^IgG^+^IgD^-^), classical MBCs (CD19^+^CD20^+^CD21^+^CD27^+^IgG^+^IgD^-^), transitional B cell (CD19^+^CD20^+^CD10^+^), and plasmablast/plasma cell (CD19^+^CD20^-^CD38^++^CD27^+/-^). For some experiments, we also stained B cells to detect expression of CD120b (clone hTNFR-M1) (BD Biosciences); CD85d (clone 42D1) and CD360 (clone 17A12) (BioLegend); FCRL3 (clone 546828) (R&D Systems); FCRL4 and FCRL5 (clone 2A6) (generously provided by M. Cooper); FCRL5 (clone 7D11) and FCRL4 (clone 1A3) (generously provided by A. Polson and Genentech Inc.). Isotype controls included mouse IgG1 (clone MOPC-21) (Tonbo Biosciences), and IgG2a (clone MOPC-173) and IgG2b (clone MPC-11) (BioLegend). Detection of mAb clones 2A6 and 1A3 was performed with rat anti-mouse IgG2a PE (clone RMG2a-62) (BioLegend), and 7D11 was detected with polyclonal goat anti-mouse IgG2b PE (Life Technologies). Confirmation of the FCRL specificities of mAb 2A6, 7D11, and 1A3 was performed using cell lines expressing recombinant *FCRL* genes (generously provided by A. Polson and Genentech Inc.) [53]. Cell lines were cultured as previously described [53], stained with mAb 2A6, 7D11, or 1A3 in the presence of Fc Block (eBioscience), and stained with the secondary antibodies described above. Human B cell FCRL staining was similar, except that cells were first stained with mAb 2A6, 7D11, 1A3, IgG2a isotype control, or mouse IgG2b isotype control in the presence of Fc block, followed by secondary antibody staining and subsequent staining for lineage markers.

### IgG ELISpot

PBMCs were stained as above and flow cytometrically sorted into the following subsets: atMBCs (CD19^+^CD20^+^CD21^-^CD27^-^IgG^+^IgD^-^), classical MBCs (CD19^+^CD20^+^CD21^+^CD27^+^IgG^+^IgD^-^), transitional (CD19^+^CD20^+^CD10^+^), and plasmablast/plasma cell (CD19^+^CD20^-^CD38^++^). To measure spontaneous antibody secretion, sorted cells were placed in 200 μl of RPMI media supplemented with 5% FBS for 18 h in ELISpot plates (Millipore) that had been coated overnight at 4°C with 10 μg/ml of goat anti-human IgG (Life Technologies). IgG-secreting cells were detected using alkaline phosphatase-conjugated goat anti-human IgG (Life Technologies) and a blue alkaline phosphatase substrate kit (Vector Laboratories). Spots were enumerated using an AID ELISpot Reader and software (AID ELISpots). To measure antibody production after stimulation, B cells were sorted based on FCRL5 expression in the atMBC and classical MBC subsets, and autologous CD3^+^ T cells were added at a 20:1 T cell to B cell ratio. Sorted cells were then stimulated with 2.5 μg/ml of CpG ODN 2006 (InvivoGen) and 2.5 μg/ml of F(ab’)_2_ goat anti-human IgG H+L chain (Jackson ImmunoResearch) for 4 d. After 4 d, cells were washed and incubated for 12 h on an ELISpot plate coated with goat anti-human IgG (Life Technologies). ELISpot plates were developed and enumerated as described above.

### Statistical analysis

Statistics for microarray analysis are described above. All other comparisons between groups utilized nonparametric Wilcoxon rank-sum or signed-rank tests for unpaired and paired comparisons, respectively. Comparisons of the percentage of atMBCs expressing FCRL5 between Nagongera and Walukuba were also evaluated using multivariate linear regression to account for potential confounding by age. A p-value of < 0.05 was considered significant.

## Supporting Information

S1 Fig Atypical memory B cell frequencies increase with age in an area of Uganda with high *P*. *falciparum* transmission. PBMCs from individuals of different age groups were labeled with antibodies to atMBCs (IgG^+^CD21^-^CD27^-^CD19^+^CD20^+^) and are reported as a percentage of total B cells excluding plasmablasts (CD19^+^CD20^+^). PBMCs from non-*P*. *falciparum* exposed US adults were also labeled with antibodies to atMBCs to establish baseline frequencies of atMBCs in the absence of *P*. *falciparum* exposure.
(EPS)

S2 Fig qPCR analysis of *FCRL* family expression in classical and atypical memory B cells. Gene expression analysis was performed by qRT-PCR as described in the methods. Relative expression of each *FCRL* gene is shown normalized to expression of *ACTB*. Statistical significance was determined using the Wilcoxon signed-rank test. *, p < 0.05.
(EPS)

S3 Fig Gating strategy for identifying memory B cell subsets. PBMCs were labeled with CD3/14, CD19, CD10, CD21, CD27, IgG and IgD to define atypical and classical MBCs to be sorted for microarray analysis. B cells were progressively gated and defined by being CD3/14^-^ (non-monocyte and T cells), CD19^+^, CD10^-^ (non-transitional B cells), with atypical and classical MBC subsets being CD21^-^CD27^-^ and CD21^+^CD27^+^, respectively, and with both memory subsets being surface IgG^+^.
(EPS)

S1 Table Genes differentially expressed between classical and atypical memory B cells. (XLSX)

S2 Table Genes identified in other contexts as being differentially expressed in nonclassical memory B cell subsets. (XLSX)
